# Optimization of Magnetoplasmonic Behavior in Ag/Fe Bilayer Nanostructures Towards Refractometric Sensing

**DOI:** 10.3390/s25051419

**Published:** 2025-02-26

**Authors:** João Pedro Miranda Carvalho, Bernardo S. Dias, Luís C. C. Coelho, José M. M. M. de Almeida

**Affiliations:** 1 Centre for Applied Photonics, INESC TEC—Institute for Systems and Computer Engineering, Technology and Science, 4150-179 Porto, Portugal; joao.p.miranda@inesctec.pt (J.P.M.C.); lcoelho@inesctec.pt (L.C.C.C.); 2 Department of Physics and Astronomy, Faculty of Sciences, University of Porto, 4169-007 Porto, Portugal; 3 Van der Waals-Zeeman Institute for Experimental Physics, Institute of Physics, University of Amsterdam, 1098 XH Amsterdam, The Netherlands; b.m.limposerradossantosdias@uva.nl; 4 School of Science and Technology, University of Trás-os-Montes and Alto Douro, 5000-801 Vila Real, Portugal

**Keywords:** optical sensors, thin films, magnetoplasmonics, transverse MOKE

## Abstract

Magneto-optic surface plasmon resonances (MOSPRs) rely on the interaction of magnetic fields with surface plasmon polaritons (SPP) to modulate plasmonic bands with magnetic fields and enhance magneto-optical activity. In the present work, a study on the magnetoplasmonic behavior of Ag/Fe bilayers is carried out by VIS-NIR spectroscopy and backed with SQUID measurements, determining the thickness-dependent magnetization of thin-film samples. The MOSPR sensing properties of Ag/Fe planar bilayers are simulated using Berreman’s matrix formalism, from which an optimized structure composed of 15 nm of Ag and 12.5 nm of Fe is obtained. The selected structure is fabricated and characterized for refractive index (RI) sensitivity, reaching 4946 RIU^−1^ and returning an effective enhancement of refractometric sensitivity after magneto-optical modulation. A new optimized and cobalt-free magnetoplasmonic Ag/Fe bilayer structure is studied, fabricated, and characterized for the first time towards refractometric sensing, to the best of our knowledge. This configuration exhibits potential for enhancing refractometric sensitivity via magneto-optical modulation, thus paving the way towards a simpler, more accessible, and safe type of RI sensor with potential applications in chemical sensors and biosensors.

## 1. Introduction

Surface plasmon resonances give rise to electromagnetic surface waves propagating along metal–dielectric interfaces, with their properties being dictated by the media at the interface through the dispersion relation of the SPP, given by

(1) $$k_{x}^{SPP}=\frac{2\pi }{\lambda }\sqrt{\frac{\epsilon_{m}\epsilon_{d}}{\epsilon_{m}+\epsilon_{d}}},$$

where λ is the free-space wavelength and $\epsilon_{m}$ and $\epsilon_{d}$ are the relative electric permittivities of the metal and dielectric layers, respectively [1]. At the resonant wavelength, the electromagnetic field is maximum at the interface and decays exponentially into each side in the normal direction, being more strongly absorbed in the metal due to higher optical losses. In the dielectric side, the electromagnetic field can propagate significant distances, raising the sensitivity of the resonant conditions with the dielectric properties of the external medium.

The sensitivity of surface plasmon resonances to the RI has been widely explored for sensing in chemical, biomedical, and food processing industries [2], offering several advantages such as label-free detection, small sample size, and real-time monitoring capacities [3]. Despite these benefits, there are still constraints to their application, namely, the high detection limits for biological samples, which lead to the necessity of sensors with increased sensitivity. Different approaches have been conducted to achieve higher sensitivities, such as the deposition of metal oxide layers like ITO [4] or TiO_2_ [5], the synthesis of nanosized or nanostructured materials, such as nanoparticles [6], nanowires [7], and nanopore arrays [8], or tailored 2D surfaces [9] that make use of the higher field coupling of localized surface plasmon resonances (LSPR) in non-continuous nanostructures [10]. Enhancing RI sensitivity has also been explored through magneto-optical modulation of magnetoplasmonic structures [11].

**Figure 1:**
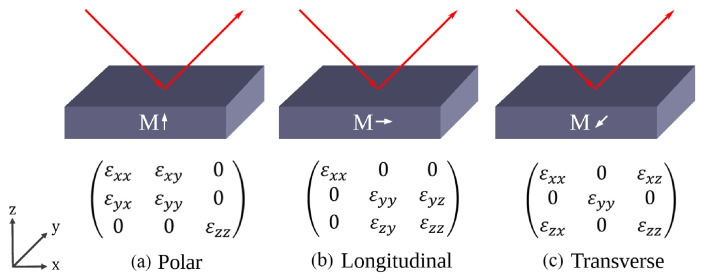
MOKE geometries and respective dielectric tensors: (**a**) polar, (**b**) longitudinal, and (**c**) transverse.

Magnetoplasmonics involves the interaction between surface plasmon resonances and magnetic materials, inducing nonreciprocal effects that enable the manipulation of plasmonic bands with magnetic fields [12]. This interaction also amplifies magneto-optic effects, namely, the magneto-optic Kerr effect (MOKE) [11]. This effect can be decomposed into three configurations based on the orientation of the magnetic field relative to the plane of the incident light: polar, longitudinal, and transverse (Figure 1).

In the case of the transverse MOKE (TMOKE), wherein magnetization is parallel to the film surface but perpendicular to the plane of the incident light, and assuming μ≈1 for the magnetic layer, only the p-polarized component of light is affected by the magnetic field while the s-polarized component remains unchanged [13]. This configuration allows for better tracking of the magneto-optical modulations as only reflectance changes occur, with no change in the polarization state of light.

The enhancement of RI sensitivity in magnetoplasmonic sensors has been achieved in multilayer structures of continuous plasmonic and magnetic metals, such as Au/Co/Au [14,15], or with ferromagnetic alloys, such as permalloy [16]. The high absorption of ferromagnetic metals led to the pursuit of magnetic garnets, such as yttrium iron garnet (YIG) and derivatives, to apply in plasmonic matrices [17,18]. The use of geometrical resonances in nanostructured media to increase RI sensitivity has also been studied, through the embedding of nanoparticles, nanoarrays [19], or nanoholes [20] of plasmonic or magnetic material in magnetoplasmonic structures.

These approaches, however, require costly materials or complex and inaccessible fabrication techniques that may be unfavorable for their widespread application. On the other hand, a single bilayer of plasmonic and ferromagnetic metals is accessible and can be made through easy, controlled fabrication with conventional thin-film deposition techniques. The use of Ag/Fe bilayers further provides the high refractometric sensitivity of Ag together with the strong ferromagnetic properties of Fe [11,12] while exhibiting fewer absorption losses than Co [21], thus enabling magnetoplasmonic behavior for sensing applications.

## 2. Magneto-Optic Surface Plasmon Resonance

The optical properties of a medium can be macroscopically described by a permittivity tensor, represented by a 3 × 3 matrix in three-dimensional space. In an isotropic medium, this matrix is diagonal, and all entries are equal. The incidence of a static magnetic field in a ferromagnetic material tends to align magnetic moments within the sample, creating a net magnetization within the magnetic layer. The effects of the magnetization can be formally represented by the insertion of off-diagonal elements in the permittivity tensor of the ferromagnetic layer that describe magneto-optical effects in the different configurations, as depicted in Figure 1. For the case of TMOKE, the off-diagonal parameter $\epsilon_{xz}$ is proportional to the magnetization **M** and the Voigt constant *V* in the following manner:

(2) $$\epsilon_{xz}=-\epsilon_{zx}=VM,$$

which couples the optical and magnetic properties of the medium, implementing the spectral dependence of magneto-optical effects in the dielectric tensor. These off-diagonal elements are usually much smaller than the diagonal part, resulting in small magneto-optical modulations of the resonance conditions of the magnetoplasmonic structure.

The change in the light–SPP coupling conditions reflects a non-reciprocity effect in SPP propagation, induced by the magnetization of a ferromagnetic layer [20]. The dispersion relation of an SPP propagating along a transversely magnetized medium is given by [22]

(3) $$k_{x}^{MOSPR}=k_{x}^{SPP}+\Delta k_{x}^{MO},$$

where $k_{x}^{SPP}$ is the SPP wave vector for a non-gyrotropic interface, as described by Equation (1), and $\Delta k_{x}^{MO}$ is the magnetization-induced modulation of the surface plasmon wave vector [11]. An exact analytical description of this term depends on the materials and respective geometry at the interface, and can be difficult to obtain. For an interface composed of a plasmonic metal and a dielectric, with a thin layer of a ferromagnetic metal at the interface, the magneto-optical modulation can be approximated as [22]

(4) $$\Delta k_{x}^{MO}\left(\epsilon_{xz}\right)\approx \frac{2t_{fm}k_{0}^{2}\epsilon_{d}^{2}\epsilon_{xz}}{-\epsilon_{m}\epsilon_{fm}}i,$$

with $t_{fm}$ being the thickness of the ferromagnetic layer, $k_{0}$ the free-space wave vector of incident light, $\epsilon_{d}$ the permittivity of the dielectric layer, $\epsilon_{m}$ the permittivity of the metal layer, and $\epsilon_{fm}$ and $\epsilon_{xz}$ the diagonal and off-diagonal entries of the permittivity tensor of the ferromagnetic layer, respectively [22].

**Figure 2:**
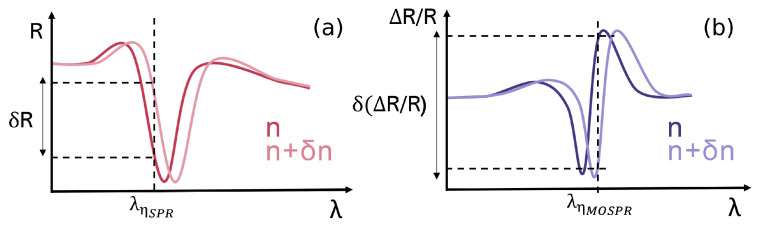
Illustration of reflectance sensitivity to the RI in (**a**) conventional plasmonic bands, and enhanced RI sensitivity for (**b**) magneto-optical modulation of magnetoplasmonic structures.

The transverse magnetization of the ferromagnetic layer, parallel to the film surface but normal to the direction of light propagation, causes no change in the polarization state of light reflecting off a magnetic film, in contrast to other possible magnetization directions [13]. The normalized MOSPR signal reflected off a magnetoplasmonic structure, combining plasmonic and magneto-optical materials, can be characterized by

(5) $$\frac{\Delta R}{R}=\frac{R(-M)-R(+M)}{R(-M)},$$

where *R*(−*M*) and *R*(+*M*) are the reflectance spectra of the magnetoplasmonic structure for negative and positive saturation magnetizations of the ferromagnetic layer, respectively [12]. A plot of Equation (5) (Figure 2b) depicts a peak in intensity due to a shift in the resonance wavelength of the SPP, which is, in turn, a direct consequence of the modulation of the dispersion relation of the SPP for transverse magnetic field incidence. This modulation of the SPR spectra can be applied in the sensing of small RI variations by monitoring the variations in the ΔR/R signal at a given interrogation wavelength for varying RIs.

The spectral sensitivity for changes in reflectance with external RI variations for SPR and MOSPR measurements [23] can be defined as, respectively,

(6) $$\eta_{SPR}=\frac{\partial R}{\partial \lambda }\frac{\partial \lambda }{\partial n},\;\mathrm{and}$$

(7) $$\eta_{MOSPR}=\frac{\partial (\Delta R/R)}{\partial \lambda }\frac{\partial \lambda }{\partial n}.$$

The interrogation wavelength for each spectrum is that of the maximum value of $\eta_{SPR}$ and $\eta_{MOSPR}$, being the wavelength of largest reflectance variation for a given RI change. For a certain set of parameters, the high slope of the MOSPR signal will result in high reflectance variations at the interrogation wavelength for an RI shift, rendering high sensitivities and increasing the performance capacity of a given structure (Figure 2).

## 3. Methodology

### 3.1. Simulation Methodology

The simulation work was developed in a Python-based numerical implementation of Berreman’s 4×4 transfer matrix method [24,25], based upon a previous version developed by our group [26], for the simulation of reflectance spectra of multilayer thin-film stacks starting from the tensorial permittivities of desired materials. The data of the diagonal permittivities for Ag and Fe layers were extracted from Ciesielski et al. [27] and Johnson et al. [21], respectively, whereas the off-diagonal permittivities accounting for the transverse magnetization of the Fe layers were extracted from Ferguson [28].

The simulation of different geometries and thicknesses of Ag and Fe, and of the RI of external media, was carried out. The MOSPR curves, as defined by Equation (5), were simulated starting from the simulated SPR spectra for symmetric saturation magnetizations +M and −M and for each RI value. For each set of SPR and MOSPR bands, the spectral reflectance sensitivity to RI variations was calculated, as defined in Equations (3) and (4), respectively. These two sensitivities were then compared and structures in which $\eta_{SPR}<\eta_{MOSPR}$ were fabricated for experimental characterization.

Owing to the different natures of the SPR and MOSPR measurements, a normalization of the signal by the respective signal-to-noise ratio (SNR) must be made for any sensitivity comparison of experimental data [23]. Such an adjustment was made by defining the minimum detectable signal, $S_{min}$, as five times the standard deviation of the five repeats taken for each measurement of the signal. The effective sensitivity adjusted for the SNR is then given by $\eta_{E}=\eta /S_{min}$, where η is either $\eta_{SPR}$ or $\eta_{MOSPR}$, as given by (3) and (4), respectively. For the simulation work, a standard deviation figure of $\sigma =3\times 10^{-4}$ was defined for both SPR and MOSPR signals based on the magnitude of σ determined from experimental measurements.

### 3.2. Experimental Methodology

The thin-film bilayers were deposited on $SiO_{2}$ substrates previously cleaned in ultrasonic baths of acetone, ethanol, and de-ionized water (Milli-Q water systems, Merck Millipore, Burlington, MA, USA) for 5 min. The thin films were fabricated by RF magnetron sputtering deposition. A base pressure of $8\times 10^{-6}$ mbar was set to remove gaseous contaminants from the sputtering chamber, preventing contamination of the deposited thin films. Before deposition, the sputtering targets of Ag (Testbourne AG-ST-2-6-4N) and Fe (Testbourne FE-ST-2-3-4N, estbourne Ltd., Basingstoke, UK) were plasma etched for 10 min to clear out contaminants from the surface of the sputtering targets. The sputtering deposition of Ag and Fe layers was carried out at 12 and 110 W, respectively, under a controlled argon pressure of $7\times 10^{-3}$ mbar.

**Figure 3:**
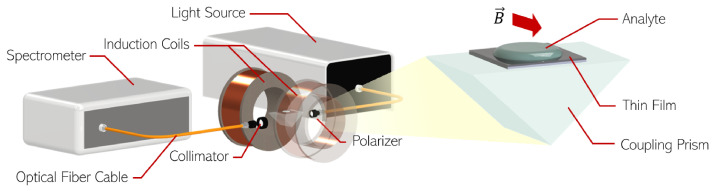
Kretschmann-based setup for optical characterization of magnetoplasmonic thin films. A broadband light source allows spectral interrogation, and two induction coils allow transverse uniform magnetic fields.

The fabricated samples were characterized by VIS-NIR spectroscopy in a modified Kretschmann configuration setup, as detailed in Figure 3. Linearly p-polarized light was incident on the prism, reflecting off the top surface at a 74° angle with the normal to the surface plane. The RI was changed by flowing water-based solutions of known RIs, measured at 589 nm using an Abbe refractometer (ATAGO DR-A1, ATAGO Co., Ltd., Tokyo, Japan). A microfluidic Teflon chamber was tightly fitted to the top of the prism for controlled analyte flow, and two induction coils were positioned at each side of the prism, inducing a transverse magnetic field with an approximately uniform profile in between them.

Identical samples were fabricated under the same experimental conditions for characterization of their magnetic properties. For the effect, a comparative study of the hysteretic behavior of the magnetoplasmonic thin films was carried out by VIS-NIR spectroscopy and with a superconducting quantum interference device (SQUID, Quantum Design, San Diego, CA, USA).

The VIS-NIR characterization was performed with the setup shown in Figure 3. Several spectra were recorded for sweeping values of DC electric current in the induction coils, measured with a benchtop digital multimeter (Hewlett Packard 34401A, Keysight, Rosa, CA, USA). The DC power supply (Univolt DT305AD, Lektronix, Aldridge, UK) enabled a maximum current of 1.6A through the induction coils connected in series ($R_{coils}$ = 28.8 Ω). The magnetic field induced by the electric current in the two induction coils was first calibrated in the region between the two coils with a Hall-effect Gaussmeter (LakeShore 421, Lake Shore Cryotronics, Westerville, OH, USA). In the vicinity of the sample, a maximum 20 mT magnetic field was attained, high enough to achieve saturation of the magnetization state of the magnetoplasmonic thin films.

Magnetic characterization by SQUID (Quantum Design MPMS3, Quantum Design, San Diego, CA, USA) allows stable and precise measurement of magnetic flux, from which a quantification of magnetic moment, *m*, within the magnetic sample can be obtained. For the characterization of the hysteresis cycles, identical samples were fabricated on smaller substrates, and were characterized at room temperature for magnetic fields ranging from −50 to 50 mT. From the magnitude of the saturation magnetic moment, the saturation magnetization, *M*, can be estimated considering the dimensions of the sample by

(8) $$M=\frac{m}{V}=\frac{m}{A\times t_{Fe}}$$

with *A* being the deposited area on the substrate and $t_{Fe}$ the expected thickness of the Fe layer.

## 4. Results and Discussion

### 4.1. Magnetic Characterization

The magnetic characterization of the magnetoplasmonic thin films was carried out for Ag/Fe bilayers with varying thicknesses of the Fe layer to study the impact of the thickness of ferromagnetic material on the magnetic response of the bilayer structure. Two identical batches of four bilayer films were deposited under the same experimental conditions, one for characterization of the hysteresis cycles by spectroscopic interrogation of the resonance wavelength, and the other for characterization of the hysteresis cycles with SQUID measurements. Each batch consisted of four thin films with 15 nm of Ag and 5, 10, 7.5, or 12.5 nm of Fe.

**Figure 4:**
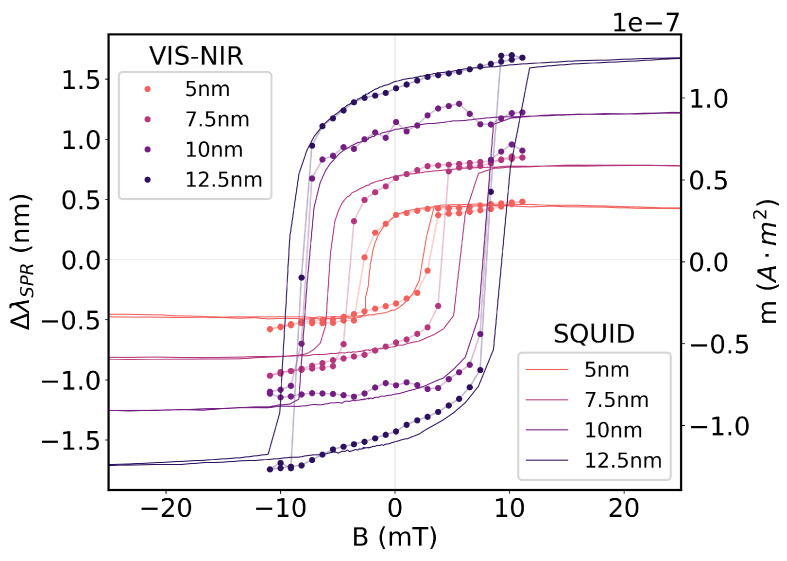
Hysteresis cycles of magnetoplasmonic Ag/Fe bilayers recorded by VIS-NIR spectroscopy and with an SQUID magnetometer.

The hysteresis cycles of each sample for each of the techniques are displayed in Figure 4. The hysteresis cycles exhibit clear ferromagnetic hysteresis and similar profiles for both characterization techniques. In both approaches, the saturation of the ferromagnetic layers occurs at proximate intensities of the magnetic field and clear remanence magnetizations are visible at B=0 for all characterized samples. The thickness dependence of the net magnetic moment is also evident, with both the net magnetic moment at saturation and the magnetic coercivity increasing for increasing thicknesses of the Fe layer. The same behavior is noticeable for the shift in the resonance wavelength of the plasmonic mode, $\Delta \lambda_{SPR}$, with larger shifts being noticeable for an increasing thickness of Fe.

It is important to note, however, that the increase in the net magnetic moment and the increase in the wavelength shift of the magnetoplasmonic bands for increasing thicknesses of Fe have different physical origins. The increase in net magnetic moment is simply related to the larger volume of ferromagnetic material, summing more contributions from individual Fe atoms for samples of increasing thickness. The increase in the wavelength shift of the magnetoplasmonic bands is more nuanced, as the order parameter governing the TMOKE is not the net magnetic moment within the sample but the magnitude of its magnetization and Voigt constant, as seen in Equation (2). The magnetization, as the net magnetic moment per unit volume, is an intrinsic property of ferromagnetic materials and its magnitude should not vary significantly with dimensions for bulk samples. In the thin-film regime, however, surface effects are significant, and their impact is higher the thinner the sample. As such, at film thicknesses below bulk dimensions (≈200 nm [29]), the magnetization increases with film thickness, leading to increased wavelength shifts of magnetoplasmonic bands. In addition to the magnetization, the Voigt constant *V* introduces non-linear spectral dependencies onto the off-diagonal component $\epsilon_{xz}$, so that the wavelength of the magnetoplasmonic resonance also has an impact on the obtained wavelength shift. These dependencies of the modulation of magnetoplasmonic bands on the thickness of ferromagnetic material are explicit in the $\Delta k_{x}^{MO}$ term of the plasmonic dispersion relation of Equation (4), where $\epsilon_{xz}$ introduces the *M* and *V* contributions, along with the thickness of the ferromagnetic layer $t_{fm}$.

**Figure 5:**
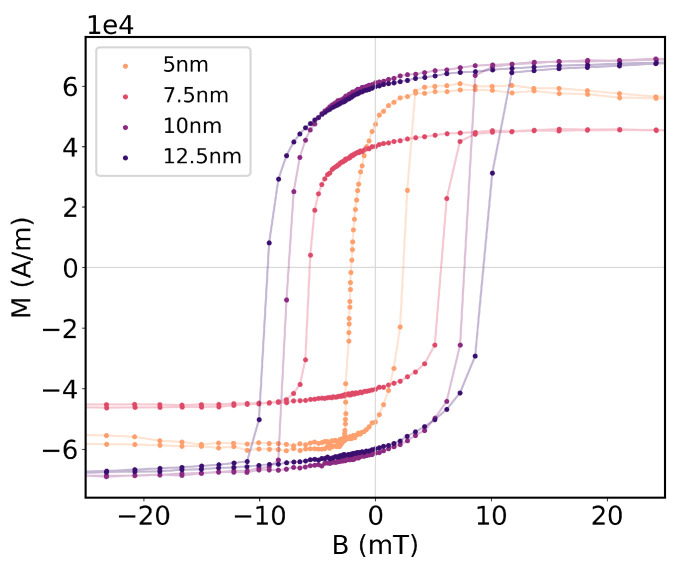
Hysteresis cycles of magnetization of magnetoplasmonic Ag/Fe bilayers measured with an SQUID magnetometer.

The net magnetizations of the thin-film samples were determined by Equation (8) and are represented in Figure 5. Some divergences were found. Although the bilayers with 10 nm and 12.5 nm exhibit the highest magnetization magnitude, it was found that the saturation magnetization for the structure with 5 nm of Fe is higher than the that of the structure with 7.5 nm of Fe. Given that the coercivity and saturation magnetic field is still higher for the bilayer with 7.5 nm of Fe, this inconsistency is likely caused by the significant errors in the estimated sample area, which greatly impact the determined magnetization magnitude. Nonetheless, the magnetic characterization backs the explicit dependence of the magneto-optical effects in the magnetization and not the incident magnetic field.

### 4.2. Magnetoplasmonic Characterization

**Figure 6:**
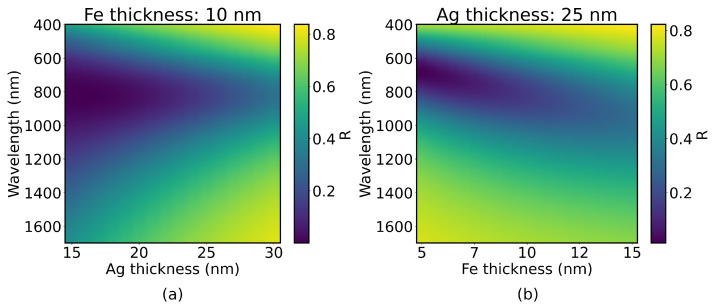
(**a**) Reflectance spectra of Ag/Fe bilayers for varying thicknesses of the Ag layer and a fixed 10 nm Fe layer; (**b**) reflectance spectra of Ag/Fe bilayers for a fixed 25 nm Ag layer and varying thicknesses of the Fe layer.

Reflectance spectra of different configurations of the Ag/Fe bilayer structure were simulated for thicknesses spanning from 15 to 30 nm for the Ag layer and 5 to 15 nm for the Fe layer. Figure 6 displays reflectance spectra for a fixed arbitrary thickness of the Fe **(a)** and Ag **(b)** layers. A larger Ag layer reflects a larger fraction of light before interacting with the magnetic Fe layer, diminishing magneto-optical activity, and smaller thicknesses will not sustain optimal plasmonic behavior, exhibiting widened bands. Similarly, larger thicknesses of Fe increase optical damping, resulting in wide bands that halt possible sensor applications, and, while smaller Fe layers do result in sharper resonances, they also significantly limit the magneto-optical response of the magnetoplasmonic bilayer.

**Figure 7:**
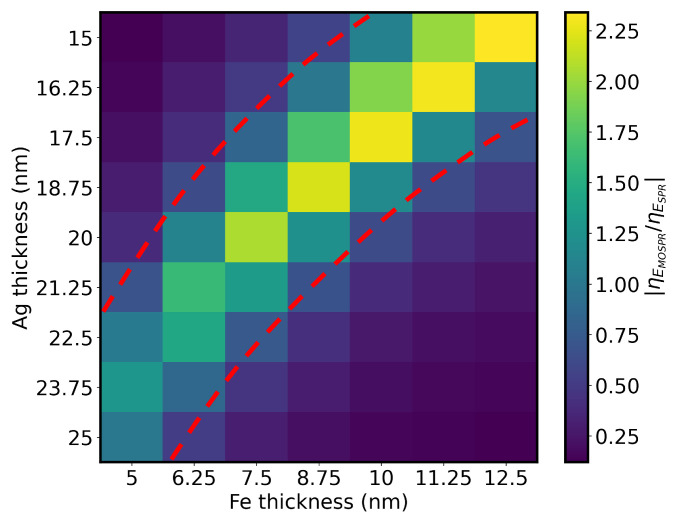
Matrix of $|\eta_{E_{MOSPR}}/\eta_{E_{SPR}}|$ ratios for different Ag/Fe bilayer thickness configurations. The configurations of the highest MOSPR sensitivity enhancement are delineated by red dashed lines.

The MOSPR bands were determined from the measured SPR spectra for opposite saturation magnetizations. Both $\eta_{SPR}$ and $\eta_{MOSPR}$ were determined and Figure 7 depicts a matrix expressing the $|\eta_{E_{MOSPR}}/\eta_{E_{SPR}}|$ ratio for the different thickness combinations of Ag/Fe bilayers, showing significant variations for slightly different deposited thicknesses. The structures with the highest ratio are in the diagonal delimited by the red dashed lines. From these, it can be seen that the structure with 15 nm of Ag and 12.5 nm of Fe has the highest magnetoplasmonic enhancement of RI sensitivity.

**Figure 8:**
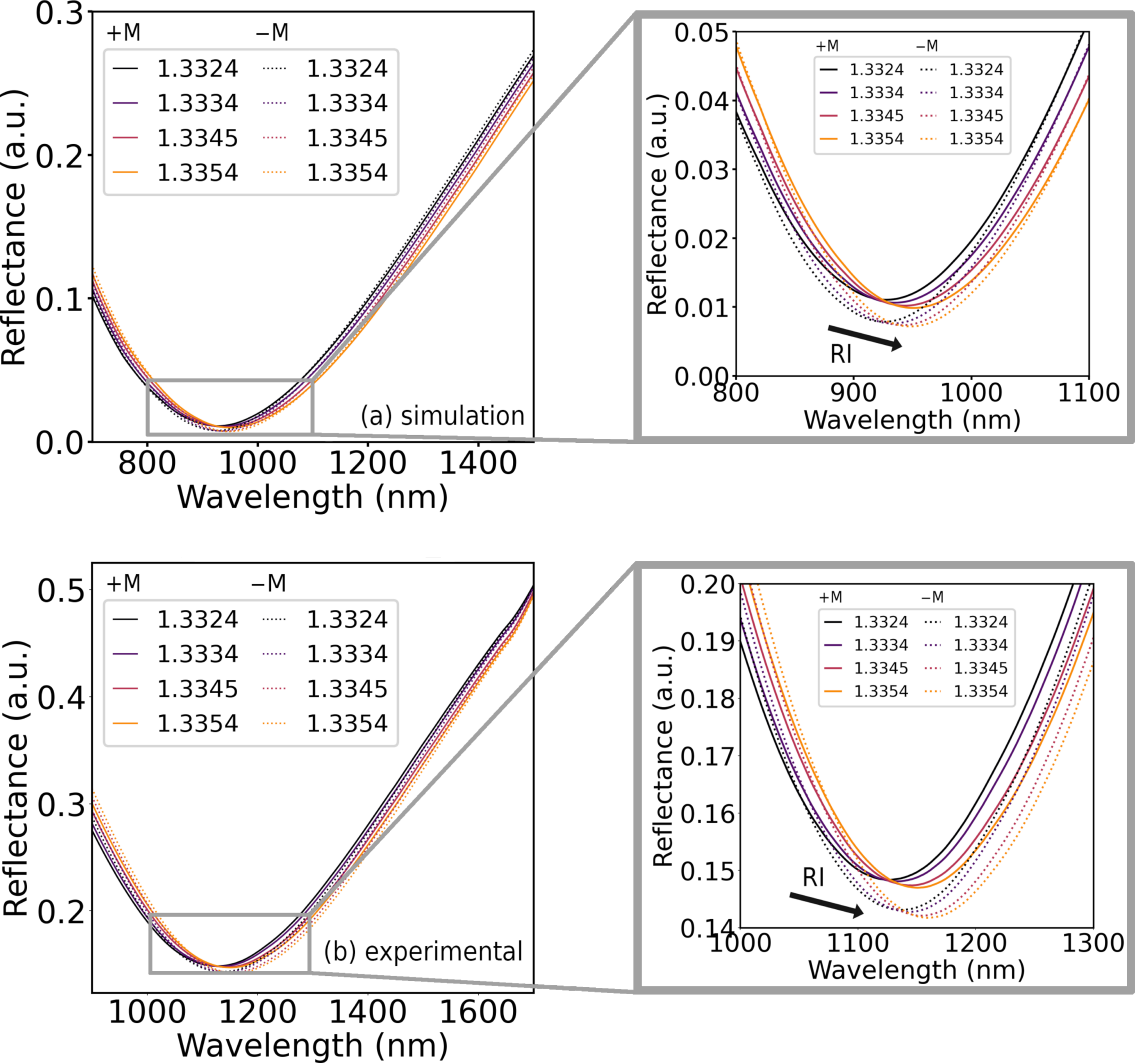
Reflectance spectra of 15 nm Ag/12.5 nm Fe bilayer for symmetric saturation magnetizations (+M continuous line, −M dotted line) and different RIs: (**a**) simulation and (**b**) experimental.

**Figure 9:**
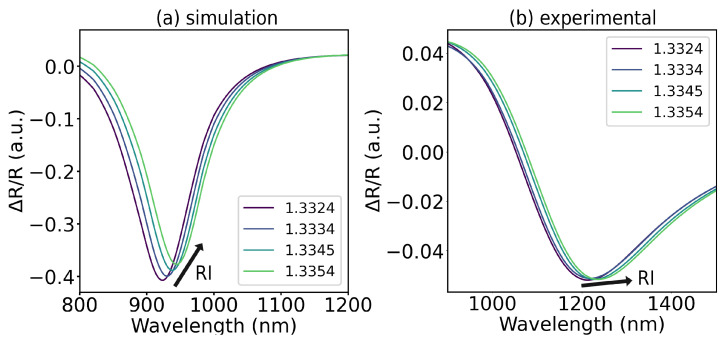
MOSPR reflectance spectra, as defined in Equation (2), of 15 nm Ag/12.5 nm Fe bilayer for symmetric saturation magnetizations and different RIs: (**a**) simulation and (**b**) experimental.

Several of the simulated structures were then fabricated and characterized. Overall, the experimental spectra and sensitivities did not rigorously match those simulated. For the 15 nm Ag/12.5 nm Fe structure, the simulated and experimental spectra fairly match, apart from a shift of resonant wavelength and an increase in the reflectance minimum, possibly due to reflections between the coupling prism and the substrate, not accounted for in TMM simulation work. The experimental spectrum exhibits a prominent magneto-optical modulation of the plasmonic bands, and an enhancement of RI sensitivity is registered. The simulated and experimental spectra for this structure are represented in Figure 8a,b, respectively, and the simulated and experimental MOSPR bands are illustrated in Figure 9a,b, respectively.

The MOSPR experimental band exhibits a magneto-optical modulation similar to the simulated band, suggesting a reasonable match in magneto-optical response, despite having a more broadened profile with a smaller magnitude than the simulated one.

**Figure 10:**
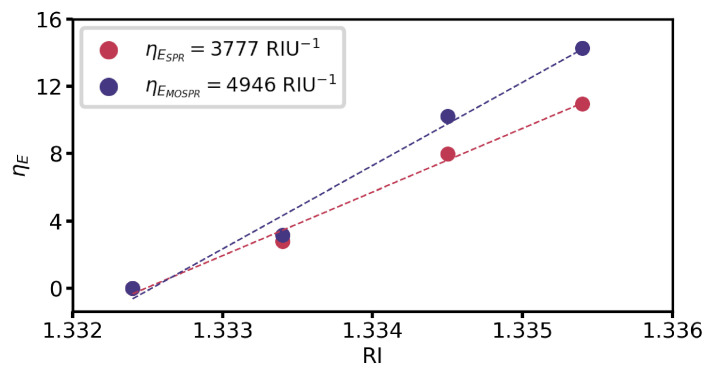
Experimental sensitivities of SPR and MOSPR spectra for 15 nm Ag/12.5 nm Fe bilayer.

These small mismatches impact the measured RI sensitivity in reflectance interrogation for SPR and MOSPR spectra, falling short of the simulated two-fold MOSPR enhancement. Nonetheless, an enhancement is still attained, with an MOSPR sensitivity enhancement of 30% over the obtained RI sensitivity for SPR interrogation, as shown in Figure 10.

The magnetoplasmonic behavior dependence on the characteristics of the fabricated structures, such as the film thickness and its optical and magnetic properties, poses a challenge in accurate simulation of magnetoplasmonic behavior. A considerable wavelength shift is noticeable for all spectra and is likely due to differences between the optical constants used for the simulation of plasmonic spectra and the actual optical properties of the fabricated structures, which can be critical for thin films under 20 nm [30,31]. The simulation of magneto-optical properties is also prone to deviations between the off-diagonal entries used in the simulation of the TMOKE in the Fe layer and those effectively observed in the fabricated samples since the magneto-optical properties and the magnetization of Fe samples depend heavily on film thickness in the thin-film regime [29]. Given the small magnitude of the magneto-optical effects, minor irregularities between the properties of simulated and fabricated samples can lead to significant errors between simulated and experimental MOSPR profiles. The measurement of the optical and magneto-optical constants of the fabricated thin films by ellipsometry, and their application in the simulation procedure, could provide a more accurate and detailed study of magnetoplasmonic structures towards refractometric sensing.

## 5. Conclusions

In the presented work, a distinct approach for visualizing hysteresis cycles in magneto-optical thin films under plasmonic enhancement was introduced and found to be useful for accessing and estimating magnetic and magnetoplasmonic properties using an accessible broadband light source. On the path towards refractometric sensing, a relative success was achieved for the simulation of magneto-optical surface waves, allowing a visualization of spectral dependencies in optical and magneto-optical effects. Some inconsistencies between simulated and experimental results arose from the different spectral dependencies of the off-diagonal elements applied in the simulations and those present in the fabricated structures of the thin films. Despite that, a structure with 15 nm of Ag and 12.5 nm of Fe attained a 30% enhancement of the sensitivity to external RI, validating the use of widely accessible, safe, and practical thin films of Fe for the enhancement of plasmonic sensitivity to the RI by magneto-optical modulation.

## Data Availability

Data underlying the results presented in this paper are not publicly available but may be obtained from the authors upon reasonable request.
